# A resource of human coronavirus protein-coding sequences in a flexible, multipurpose Gateway Entry clone collection

**DOI:** 10.1093/g3journal/jkad105

**Published:** 2023-06-02

**Authors:** Benjamin Weller, Chung-Wen Lin, Oxana Pogoutse, Mayra Sauer, Nora Marin-de la Rosa, Alexandra Strobel, Veronika Young, Jennifer J Knapp, Ashyad Rayhan, Claudia Falter, Dae-Kyum Kim, Frederick P Roth, Pascal Falter-Braun

**Affiliations:** Institute of Network Biology (INET), Molecular Targets and Therapeutics Center (MTTC), Helmholtz Zentrum München, German Research Center for Environmental Health, Ingolstädter Landstr. 1, 85764 Munich-Neuherberg, Germany; Institute of Network Biology (INET), Molecular Targets and Therapeutics Center (MTTC), Helmholtz Zentrum München, German Research Center for Environmental Health, Ingolstädter Landstr. 1, 85764 Munich-Neuherberg, Germany; Donnelly Centre for Cellular and Biomolecular Research (CCBR), University of Toronto, 160 College St, Toronto, Ontario M5S 3E1, Canada; Department of Molecular Genetics, University of Toronto, 1 King's College Cir., Toronto, Ontario M5S 1A8, Canada; Lunenfeld-Tanenbaum Research Institute (LTRI), Sinai Health System, 600 University Avenue, Toronto, Ontario M5G 1X5, Canada; Center for Cancer Systems Biology (CCSB), Dana-Farber Cancer Institute, 450 Brookline Ave, Boston, MA 02215, USA; Institute of Network Biology (INET), Molecular Targets and Therapeutics Center (MTTC), Helmholtz Zentrum München, German Research Center for Environmental Health, Ingolstädter Landstr. 1, 85764 Munich-Neuherberg, Germany; Institute of Network Biology (INET), Molecular Targets and Therapeutics Center (MTTC), Helmholtz Zentrum München, German Research Center for Environmental Health, Ingolstädter Landstr. 1, 85764 Munich-Neuherberg, Germany; Institute of Network Biology (INET), Molecular Targets and Therapeutics Center (MTTC), Helmholtz Zentrum München, German Research Center for Environmental Health, Ingolstädter Landstr. 1, 85764 Munich-Neuherberg, Germany; Institute of Network Biology (INET), Molecular Targets and Therapeutics Center (MTTC), Helmholtz Zentrum München, German Research Center for Environmental Health, Ingolstädter Landstr. 1, 85764 Munich-Neuherberg, Germany; Donnelly Centre for Cellular and Biomolecular Research (CCBR), University of Toronto, 160 College St, Toronto, Ontario M5S 3E1, Canada; Department of Molecular Genetics, University of Toronto, 1 King's College Cir., Toronto, Ontario M5S 1A8, Canada; Lunenfeld-Tanenbaum Research Institute (LTRI), Sinai Health System, 600 University Avenue, Toronto, Ontario M5G 1X5, Canada; Center for Cancer Systems Biology (CCSB), Dana-Farber Cancer Institute, 450 Brookline Ave, Boston, MA 02215, USA; Donnelly Centre for Cellular and Biomolecular Research (CCBR), University of Toronto, 160 College St, Toronto, Ontario M5S 3E1, Canada; Department of Molecular Genetics, University of Toronto, 1 King's College Cir., Toronto, Ontario M5S 1A8, Canada; Lunenfeld-Tanenbaum Research Institute (LTRI), Sinai Health System, 600 University Avenue, Toronto, Ontario M5G 1X5, Canada; Center for Cancer Systems Biology (CCSB), Dana-Farber Cancer Institute, 450 Brookline Ave, Boston, MA 02215, USA; Institute of Network Biology (INET), Molecular Targets and Therapeutics Center (MTTC), Helmholtz Zentrum München, German Research Center for Environmental Health, Ingolstädter Landstr. 1, 85764 Munich-Neuherberg, Germany; Donnelly Centre for Cellular and Biomolecular Research (CCBR), University of Toronto, 160 College St, Toronto, Ontario M5S 3E1, Canada; Department of Molecular Genetics, University of Toronto, 1 King's College Cir., Toronto, Ontario M5S 1A8, Canada; Lunenfeld-Tanenbaum Research Institute (LTRI), Sinai Health System, 600 University Avenue, Toronto, Ontario M5G 1X5, Canada; Center for Cancer Systems Biology (CCSB), Dana-Farber Cancer Institute, 450 Brookline Ave, Boston, MA 02215, USA; Department of Cancer Genetics and Genomics, Roswell Park Comprehensive Cancer Center, Elm & Carlton Streets, Buffalo, NY 14263, USA; Donnelly Centre for Cellular and Biomolecular Research (CCBR), University of Toronto, 160 College St, Toronto, Ontario M5S 3E1, Canada; Department of Molecular Genetics, University of Toronto, 1 King's College Cir., Toronto, Ontario M5S 1A8, Canada; Lunenfeld-Tanenbaum Research Institute (LTRI), Sinai Health System, 600 University Avenue, Toronto, Ontario M5G 1X5, Canada; Center for Cancer Systems Biology (CCSB), Dana-Farber Cancer Institute, 450 Brookline Ave, Boston, MA 02215, USA; Department of Computer Science, University of Toronto, 40 St. George Street, Toronto, Ontario M5S 2E4, Canada; Institute of Network Biology (INET), Molecular Targets and Therapeutics Center (MTTC), Helmholtz Zentrum München, German Research Center for Environmental Health, Ingolstädter Landstr. 1, 85764 Munich-Neuherberg, Germany; Microbe-Host Interactions, Faculty of Biology, Ludwig-Maximilians-Universität (LMU) München, Großhaderner Str. 9, 82152 Planegg-Martinsried, Germany

**Keywords:** coronavirus, HCoV, coding sequence, SARS-CoV-2, OC43, MERS, HKU1, 229E, NL63, Gateway Entry clone

## Abstract

The COVID-19 pandemic has catalyzed unprecedented scientific data and reagent sharing and collaboration, which enabled understanding the virology of the SARS-CoV-2 virus and vaccine development at record speed. The pandemic, however, has also raised awareness of the danger posed by the family of coronaviruses, of which 7 are known to infect humans and dozens have been identified in reservoir species, such as bats, rodents, or livestock. To facilitate understanding the commonalities and specifics of coronavirus infections and aspects of viral biology that determine their level of lethality to the human host, we have generated a collection of freely available clones encoding nearly all human coronavirus proteins known to date. We hope that this flexible, Gateway-compatible vector collection will encourage further research into the interactions of coronaviruses with their human host, to increase preparedness for future zoonotic viral outbreaks.

## Introduction

The global COVID-19 pandemic, caused by severe acute respiratory syndrome coronavirus 2 (SARS-CoV-2), and the outbreaks of severe acute respiratory syndrome coronavirus (SARS-CoV-1) and Middle East respiratory syndrome-related coronavirus (MERS-CoV) in 2003 and 2012, respectively, have demonstrated the potential hazards that coronaviruses pose to the human population and society. Infection with any of these viruses can lead to diffuse alveolar damage, symptoms of pneumonia, and acute respiratory distress syndrome (Zhu *et al*. 2020; Bösmüller *et al*. 2021), with case fatality rates for SARS-CoV-1 and MERS-CoV of ∼10% and ∼35%, respectively (de Wit *et al*. 2016; Zhu *et al*. 2020). While infection with SARS-CoV-2 is less lethal overall (Zhu *et al*. 2020), its high transmissibility (Petersen *et al*. 2020) and rapid global spread (WHO, COVID-19 Weekly Epidemiological Update Edition 45, published 22 June 2021) have taken an immense human toll and had a dramatic impact on public and social life worldwide.

Until the year 2000, only 2 zoonotic coronaviruses [human coronaviruses, (HCoVs)] were known, HCoV-OC43 and HCoV-229E, which cause mild respiratory symptoms upon infection and have likely crossed the species barrier to humans 150–200 years ago (Forni *et al*. 2017). Two additional mildly pathogenic HCoVs were discovered in 2004 and 2005 from clinical samples, HCoV-NL63 (van der Hoek *et al*. 2004) and HCoV-HKU1 (Fouchier *et al*. 2004). However, recent studies have identified a plethora of genetically diverse coronaviruses, which circulate predominantly in wild animals. The abundance of these coronaviruses serves as a potential reservoir for similar zoonotic pandemics to occur in the future (Li *et al*. 2005; Menachery *et al*. 2015; Menachery *et al*. 2016; Hu *et al*. 2017; Boni *et al*. 2020).

Whereas vaccines against SARS-CoV-2 have been approved, vaccines against the other coronaviruses do not exist to date. Moreover, due to the time required for vaccine development, viral evolution, and incomplete vaccination rates, small molecule–based treatments are still a key component in fighting infections. To this day, effective treatments against SARS-CoV-2–induced Coronavirus Disease 2019, as well as the acute respiratory distress syndrome linked to infection with SARS-CoV-1, SARS-CoV-2, and MERS-CoV, are scarce. A continued and concerted research effort is therefore necessary in order to understand the molecular mechanisms by which these viruses undermine the health and immune responses of their host. The molecular function of individual viral protein-coding sequences is an essential part towards this goal, and systematically identifying targeted host factors has been productive in the discovery of potential drug targets (Pfefferle *et al*. 2011; Gordon *et al*. 2020a; Gordon *et al*. 2020b; Li *et al*. 2021; Stukalov *et al*. 2021).

In order to facilitate and streamline these efforts, we have assembled a comprehensive collection of Entry clones, comprising the majority of protein-coding sequences [here also referred to as viral open reading frames (vORFs)] from the 7 HCoVs currently known to infect humans. Entry clone vectors are part of the Gateway cloning system, wherein the sequence from the Entry vector contained within specific recognition sites (attL1 and attL2) can be transferred in a site- and direction-specific manner in a 1-step recombination reaction to various Destination vectors to generate Expression vectors for downstream applications. This versatile resource should enable diverse research into the common, as well as specific functions of orthologous viral protein-coding sequences, and shed light on the distinct features that lead to the severe pathology of some HCoVs. We are confident that this will greatly expedite the identification of treatment options against similar dangerous zoonoses in the future. Compatibility with the Gateway system allows for efficient transfer of this clone collection to a large selection of destination vectors, for expression in mammalian cells, *Saccharomyces* or *Escherichia coli*, to conduct biochemical and structural studies, to name just a few potential applications. We hope that broad public availability of this clone collection, through dissemination by Addgene, will encourage and facilitate much needed research to better understand molecular interactions of coronaviruses with its host in the viral life cycle, as well as in their modulations of host cellular, metabolic, and immune processes.

## Materials and methods

### Assembly of viral protein-coding sequences

We generated a list of viral protein-coding sequences of 7 HCoVs for synthesis at Twist Bioscience (San Francisco, CA, USA). For SARS-CoV-2, the reference sequence from the isolate Wuhan-Hu-1 (NC_045512.2) and the genome annotation from Wu *et al*. (Wu *et al*. 2020a) was used. Sequences and genome annotation for the other 6 viruses were downloaded from the NCBI database under the following accession numbers: SARS-CoV-1: NC_004718.3; MERS-CoV: NC_019843.3; HCoV-229E: NC_002645.1; HCoV-OC43: NC_006213.1; HCoV-NL63: NC_005831.2; and HCoV-HKU1: NC_006577.2. In total, 163 protein-coding sequences were identified. Twist Bioscience was not able to synthesize HCoV-229E-NSP3, HCoV-OC43-NSP3, HCoV-HKU1-NSP3, HCoV-NL63-NSP2, HCoV-HKU1-NSP4, HCoV-HKU1-NSP14, and MERS-CoV-NSP13, due to highly repetitive sequence elements and highly variable GC content. Thus, we obtained synthesis products for 156 protein-coding sequences across the 7 viruses. The HCoV-HKU1–hemagglutinin esterase coding sequence was codon optimized for *Saccharomyces cerevisiae*, due to areas with low GC content and repetitive sequence elements, to facilitate its synthesis. All remaining sequences were left unchanged, except their native start and stop codons removed, if present.

We added multipurpose linkers to all synthesis products directly 5´ and 3´ of all coding sequences. Since NSP2-16 are proteolytic cleavage products and lack a native translational start codon, we included a translational start codon in frame with the coding sequence in the 5´ linker, flanked by BamHI restriction sites for easy removal. An in-frame stop codon was added in the 3´ linker, nested between PacI and AsiSI restriction sites. SfiI restriction sites are also present in each linker at both extremities for cloning into pENTR223.1*SfiI (Invitrogen, NIH Mammalian Gene Collection program).

### Cloning of coding sequences into Gateway-compatible Entry vector

Coding sequences < 1,700 bp were synthesized as linear DNA fragments, and larger sequences incorporated as vector inserts at Twist Bioscience. For insertion of linear DNA into pENTR223.1*SfiI, the empty vector, as well as synthesized DNA products, was digested with SfiI (NEB Cat# R0123) for 1 hour at 50°C in separate reactions. The vector backbone was subsequently isolated and purified by gel electrophoresis and viral coding sequences purified using magnetic beads (Steinbrenner Laborsysteme, Cat# MDKT00010075). Vector backbone and viral DNA were then incubated together in the presence of T4 ligase (Life Technologies, Cat# EL0011), and the reaction products transformed into chemically competent *E. coli* (DH5α, in-house). Large coding sequences were released from the vector backbone by SfiI digest, purified by gel electrophoresis, ligated into SfiI–digested pENTR223.1*SfiI, and subsequently transformed. Coding sequences above 5 kbp could not be synthesized in 1 piece and were therefore split into 2 fragments, as vector inserts, with ApaLI, PluTI, XbaI, or SacI restriction sites added at the inner sides of both fragments. These synthesis products were additionally digested with the respective enzyme prior to digestion with SfiI, and both purified fragments were added to the ligation reaction. All coding sequences of SARS-CoV-1 were also split into 2 fragments with BsaI restriction sites at the inner sides of the fragments. These linear DNA synthesis products were treated with T4 polynucleotide kinase (Life Technologies, Cat# EK0032) in the presence of ATP and subsequently inserted into p4-P1R (Life Technologies), which had been digested with EcoRV and XmnI, followed by dephosphorylation with alkaline phosphatase (NEB, Cat# M0371S), by blunt-end ligation. The inserts were then released by digestion with BsaI and SfiI, purified by gel electrophoresis and the 2 fragments ligated into SfiI-digested pENTR223.1*SfiI.

Each bacterial clone with viral coding sequence from pENTR223.1*SfiI was isolated from a single colony, and the correct insert size was confirmed by PCR with the primers M13F and M13R. Additionally, all SARS-CoV-2 constructs were analyzed by full Sanger sequencing of vector inserts and all constructs subjected to NGS sequencing in a partnership between SeqWell (Beverley, MA, USA) and Addgene (Watertown, MA, USA). This revealed a divergence from the intended synthesis products for 12 constructs, 11 of which were subsequently reverted to the correct sequence by mutation PCR on the respective Entry plasmids. Supplementary Table 1 lists the 155 clones successfully generated by this method.

### Generating a clone collection without any linker regions

Because linkers added upstream and downstream of each vORF might not be compatible with some experimental designs, we further constructed a set of 201 modified entry clones in which we removed these linkers for vORFs excluding those of SARS-CoV-2 (Supplementary Table 2). The clones for SARS-CoV-2 protein-coding sequences without any linkers, in a codon-optimized version, are described elsewhere (Supplementary Table 2) (Kim *et al*. 2020). For this, each viral coding sequence on parental pENTR223.1*SfiI plasmid was amplified by PCR reaction with a forward primer containing attB sequence followed by start codon and gene-specific sequence and reverse primer consisting of attB sequence and stop codon (for those constructs with a stop codon) followed by gene-specific sequence (Supplementary Table 2). Gel-purified PCR product was inserted into pDONR223 (Wiemann *et al*. 2016) by BP reaction (Thermo Fisher, Cat# 11789100) and successful subcloning was further confirmed by Sanger sequencing (The Centre for Applied Genomics, Toronto, Canada). To this end, we generated a library of 101 viral clones without stop codons and 100 clones with a stop codon (collectively covering 85% of the 118 viral coding sequences for which cloning was attempted) for SARS-CoV-1, MERS-CoV, HCoV-HKU1, HCoV-NL63, HCoV-OC43, and HCoV-229E coronaviruses (Supplementary Table 2).

## Results and discussion

The coronavirus family is distinguished by a large, single-stranded RNA genome of ∼27–32 kb. It encodes 2 overlapping polyproteins in the 5´ region, pp1a and pp1ab, which are each proteolytically cleaved to yield a total of 16 nonstructural proteins (NSP1-16), whereas the presence of a short, putative NSP11 at the junction between pp1a and pp1ab is uncertain and not included in most HCoV genome annotations. The 3´ region harbors the structural genes encoding spike (S), nucleocapsid (N), membrane (M), and envelope (E) proteins, as well as a variable number of accessory proteins, depending on the particular virus species.

**Fig. 1.**
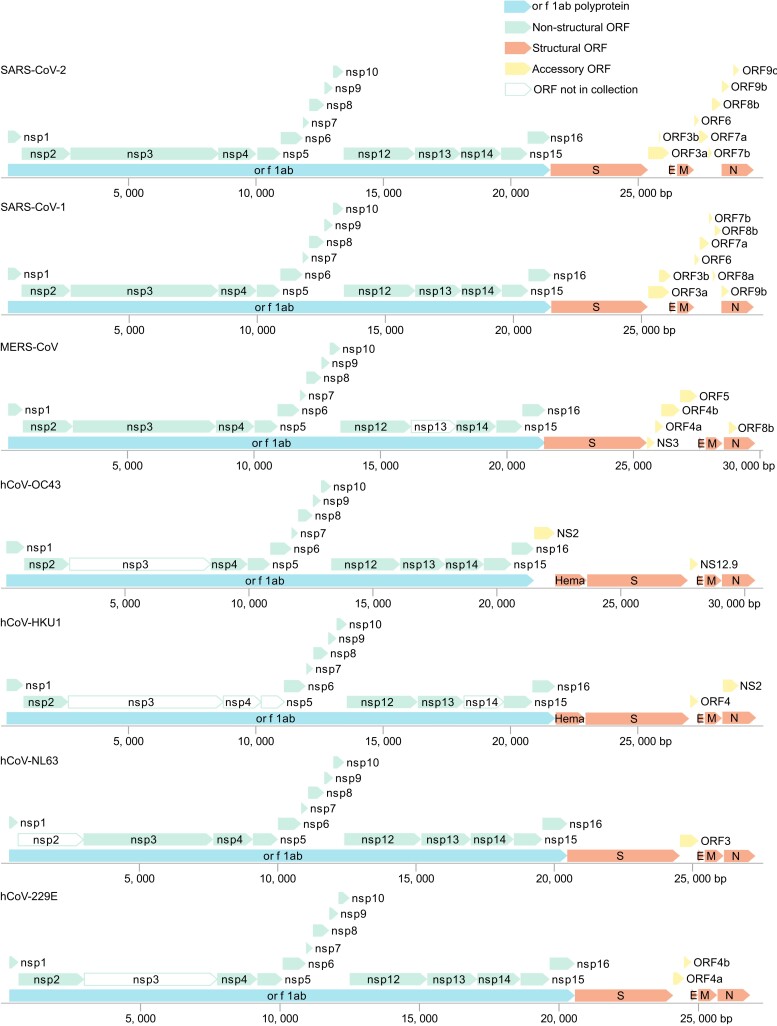
ORF structure and clone availability across the 7 HCoV genomes. Annotated ORFs from 7 HCoV genomes according to the NCBI accession numbers: SARS-CoV-2: NC_045512.2; SARS-CoV-1: NC_004718.3; MERS-CoV: NC_019843.3; HCoV-OC43: NC_006213.1; HCoV-HKU1: NC_006577.2; HCoV-NL63: NC_005831.2; and HCoV-229E: NC_002645.1. ORFs are drawn to scale based on nucleotide length (bp) indicated below each genome. ORFs colored in green (nsp1 - nsp16) are the proteolytic cleavage products from pp1a/pp1ab (orf1ab, blue) and are available as individual clones. ORFs colored in red (S, E, M, N, Hema) and yellow (NS2 - NS12.9, ORF3 - ORF9) represent structural and accessory ORFs, respectively, and are all present in the collection. ORFs drawn as open boxes are not available in the collection.

**Fig. 2.**
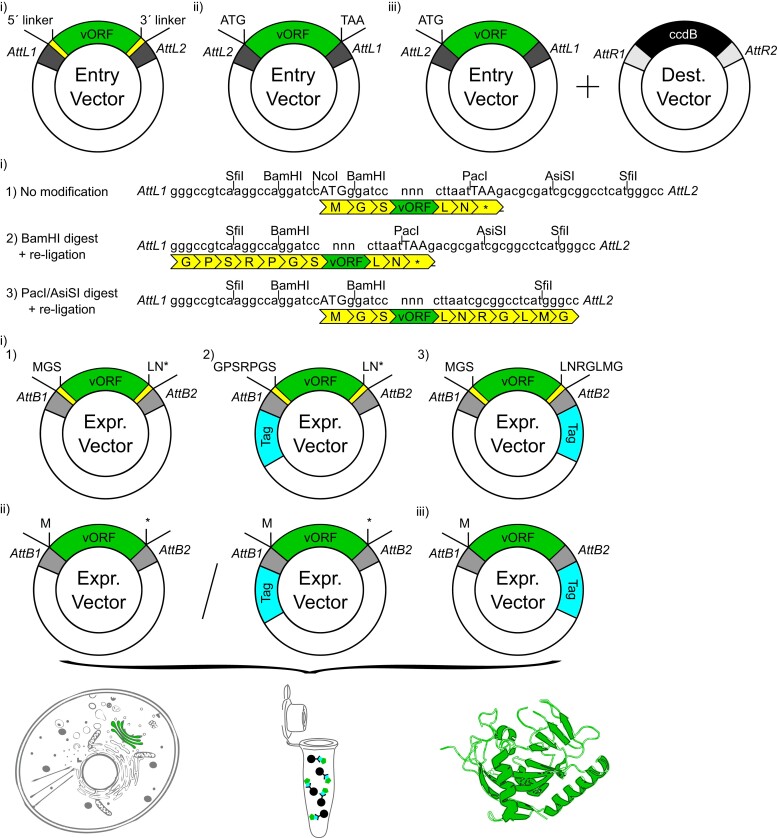
Format of Gateway Entry clones in 3 configurations, possible expression vectors to be generated, and potential downstream applications. Configuration of Entry vectors including i) 5´ and 3´ linkers, ii) native sequence “open” configuration without stop codon, and ii) native sequence “closed” configuration with stop codon. For i), the nucleotide and amino acid sequence added by the linkers is shown for the 1) unmodified, 2) BamHI–digested, and 3) PacI/AsiSI–digested Entry vectors. Possible Expression vectors generated by LR recombination reaction with an appropriate Destination vector for all configurations of Entry clones. The amino acid sequence added to expression clones from the different configurations is indicated. Potential downstream applications for the study of viral proteins using this clone collection are depicted.

The present work describes the generation of a Gateway Entry clone collection comprising nearly all protein-coding sequences contained within the genomes of all HCoVs known to date, with the cleavage products of polyproteins pp1a and pp1ab treated as individual sequences (NSP1-16, excluding a putative NSP11). In total, 155 out of 163 viral coding sequences from 7 HCoVs are included in this collection (Fig. 1). Three configurations of these constructs were generated. (1) All 155 coding sequences are available containing multipurpose 5´ and 3´ linkers (Fig. 2). Specifically, this comprises 27 annotated coding sequences of SARS-CoV-2, 27 coding sequences of SARS-CoV-1, 23 of 24 coding sequences from MERS-CoV, 18 of 22 from HCoV-HKU1, 19 of 20 from HCoV-NL63, 21 of 22 from HCoV-OC43, and 20 of 21 from HCoV-229E (Fig. 1, Supplementary Table 1). (2) A native sequence “open” configuration without stop codon and (3) a native sequence “closed” configuration with stop codon (Fig. 2) were additionally generated for 101 and 100 constructs, respectively (Supplementary Table 2). These configurations for SARS-CoV-2 protein-coding sequences, in a codon-optimized version, are described elsewhere (Supplementary Table 2) (Kim *et al*. 2020). Supplementary Table 2 lists the clones available in the open and closed native-sequence configurations.

The multipurpose 5´ and 3´ linkers were designed to allow for flexible translation start and termination to generate N-terminal or C-terminal fusion constructs in Gateway Destination plasmids by LR recombination reactions. Removal of alternative start or stop codons from 1 clone can be accomplished by digestion with BamHI (ATG) or PacI and AsiSI (STOP), respectively, and subsequent religation. Successful removal of the start codon in the 5′ linker can be confirmed by NcoI digest, since this restriction site is eliminated within the linker. Similarly, removal of the stop codon within the 3′ linker can be confirmed by PacI or AsiSI digest. The amino acids added to the coding sequences by the linkers with and without digestion, as well as the format of the constructs in the native sequence configurations, are indicated in Fig. 2.

We demonstrated the power of our ORFeome resource by generation and extensive validation of a large-scale map of the SARS-CoV-2 human protein contact interactome (Kim *et al*. 2023). For this study, ORFs for SARS-CoV-2 were transferred into five different Expression plasmids for interactome screening, validation, and functional follow-up assays (Kim *et al*. 2023). A corresponding Y2H screen using the entire clone collection is currently ongoing.

The Gateway-compatible clones facilitate a wide variety of applications, including, but not limited to, studies on subcellular localization, structure analysis, protein interaction (Fig. 2), and development of antiviral reagents. We note that this collection consists of Entry clones to enable facile recombinational subcloning of viral coding sequences into an appropriate Destination vector for downstream applications (Fig. 2). We have not quantitatively assessed individual constructs in terms of expression or function in different systems. However, the generation of a high-quality, orthogonally validated Y2H interaction data set comprising the SARS-CoV-2 subset of clones suggests that this clone collection will provide a valuable resource.

All clones described herein have been deposited to the nonprofit organization Addgene for broad dissemination. The clones can also be obtained from the authors, where acquisition from Addgene is not possible. We hope that public availability of this collection will facilitate and expedite research into structural features of HCoV proteins (Krafcikova *et al*. 2020; Thoms *et al*. 2020) and their individual molecular functions (Miorin *et al*. 2020; Wu *et al*. 2020b), with the goal of identifying potential therapeutic targets and corresponding pan-coronaviral treatments.

## Supplementary Material

jkad105_Supplementary_Data

## Data Availability

All plasmids reported herein have been deposited at the nonprofit organization Addgene for dissemination and can be obtained from the authors upon request when acquisition through Addgene is not possible. All data from the study are included in the manuscript and associated files. Supplemental material available at G3 online.
