# Supplementary material for: A resource of human coronavirus protein-coding sequences in a flexible, multipurpose Gateway Entry clone collection
Source: G3 (Bethesda). 2023 Jun 2;13(7):jkad105. doi: 10.1093/g3journal/jkad105 (PMC10320145; doi:10.1093/g3journal/jkad105)
Supplement: jkad105_Supplementary_Data [file jkad105_supplementary_data.zip › Supplemental_Material_Legends_G3-2023-404132.docx]

**SUPPLEMENTARY TABLES**

**Table S1.** List of available HCoV protein coding sequences in this collection including 5´ and 3´ linkers in the Gateway Entry vector pENTR223.1*SfiI. The reference genome accession number and coding sequence, as well as the putative function of individual proteins, is indicated. Column I lists the individual Addgene plasmid IDs. Go to https://addgene.org/”plasmid ID#” for direct access to a particular plasmid.

**Table S2.** List of available HCoV protein coding sequences in a native sequence configuration including a translational start site in the Gateway Entry vector pENTR223. Columns I and J indicate, respectively, whether the coding sequence is available with or without a stop codon. A listed Addgene plasmid ID indicates available clones. Go to https://addgene.org/”plasmid ID#” for direct access to a particular plasmid. N/A: Plasmid not available. The primer sequences used for cloning of the different configurations are listed.
